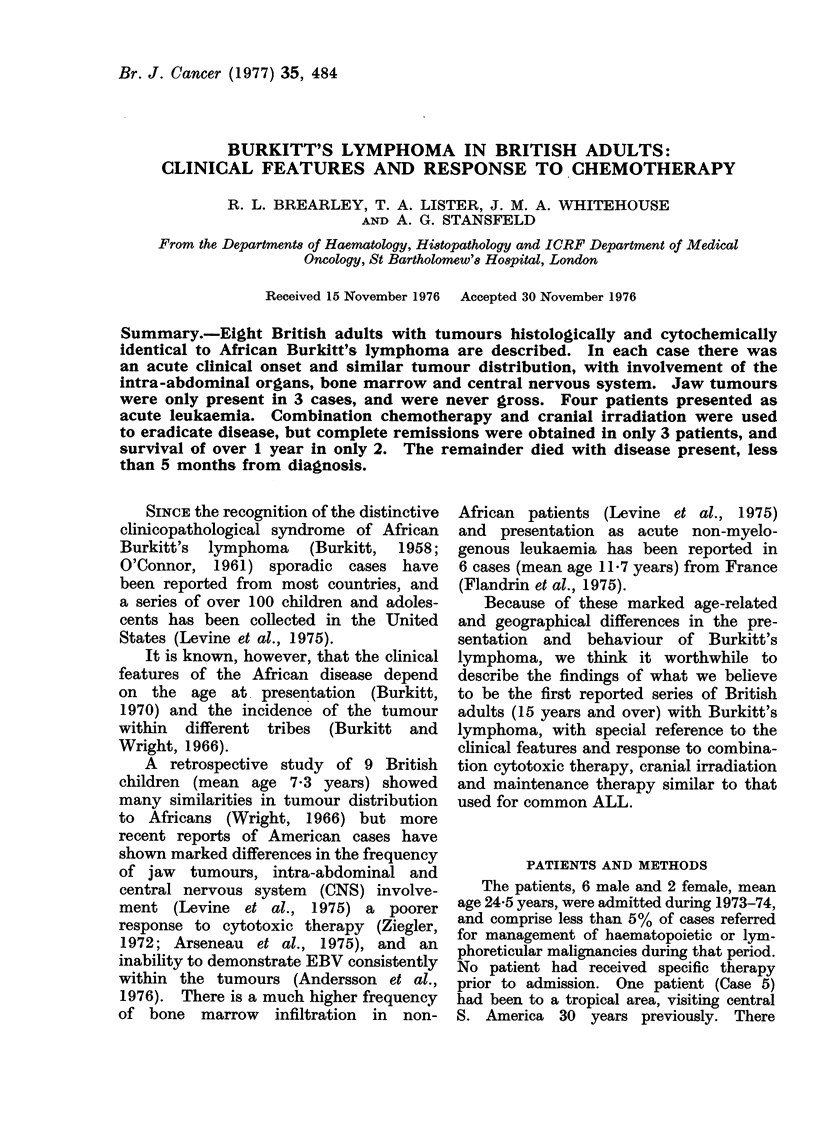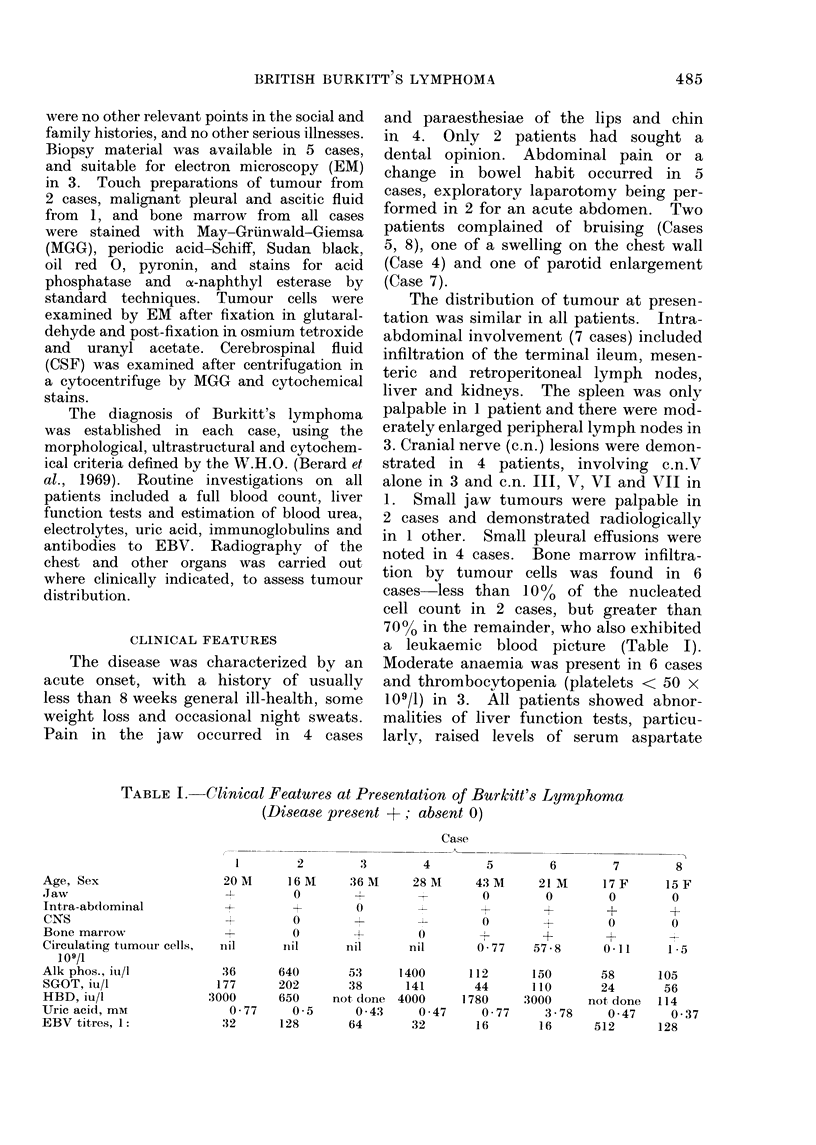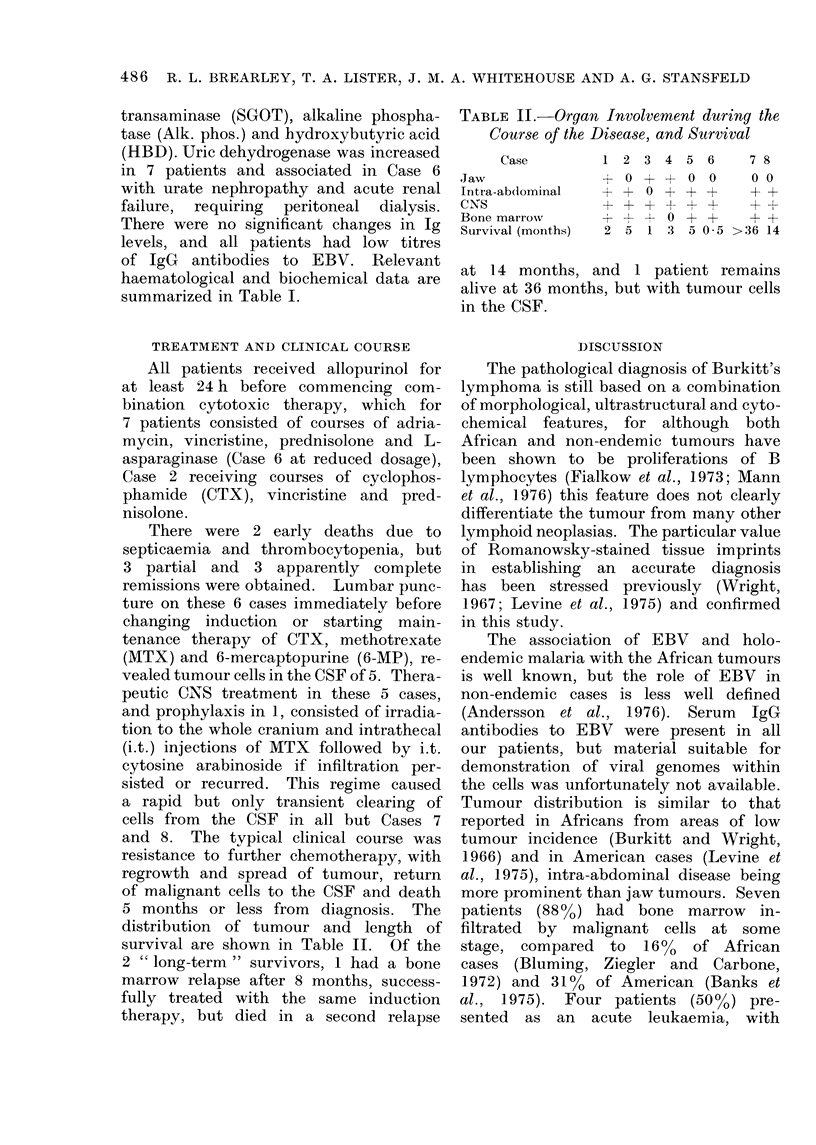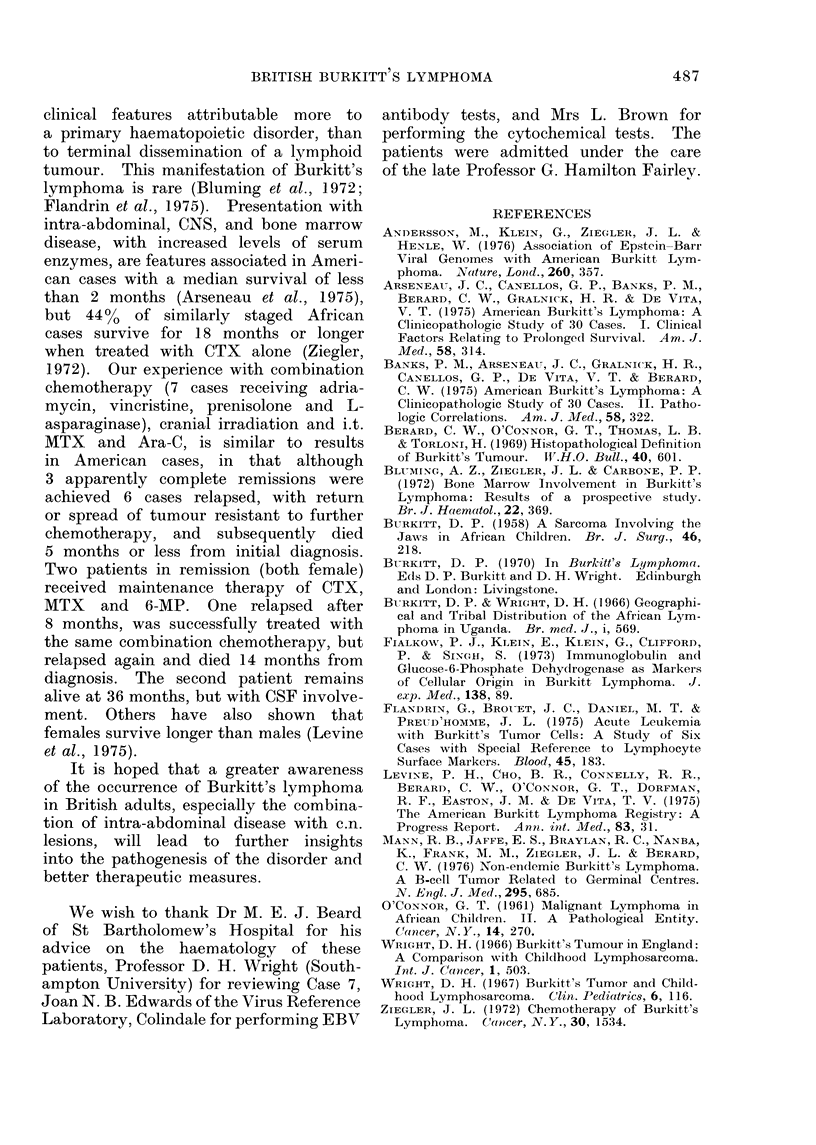# Burkitt's lymphoma in British adults: clinical features and response to chemotherapy.

**DOI:** 10.1038/bjc.1977.72

**Published:** 1977-04

**Authors:** R. L. Brearley, T. A. Lister, J. M. Whitehouse, A. G. Stansfeld

## Abstract

Eight British adults with tumours histologically and cytochemically identical to African Burkitt's lymphoma are described. In each case there was an acute clinical onset and similar tumour distribution, with involvement of the intra-abdominal organs, bone marrow and central nervous system. Jaw tumours were only present in 3 cases, and were never gross. Four patients presented as acute leukaemia. Combination chemotherapy and cranial irradiation were used to eradicate disease, but complete remissions were obtained in only 3 patients, and survival of over 1 year in only 2. The remainder died with disease present, less than 5 months from diagnosis.


*Br. J. Cancer* (1977) **35**, 484

### BURKITT'S LYMPHOMA IN BRITISH ADULTS: CLINICAL FEATURES AND RESPONSE TO CHEMOTHERAPY

R. L. BREARLEY, T. A. LISTER, J. M. A. WHITEHOUSE AND A. G. STANSFELD

*From the Departments of Haematology, Histopathology and ICRF Department of Medical Oncology, St Bartholomew's Hospital, London*

Received 15 November 1976 Accepted 30 November 1976

**Summary.—Eight British adults with tumours histologically and cytochemically identical to African Burkitt's lymphoma are described. In each case there was an acute clinical onset and similar tumour distribution, with involvement of the intra-abdominal organs, bone marrow and central nervous system. Jaw tumours were only present in 3 cases, and were never gross. Four patients presented as acute leukaemia. Combination chemotherapy and cranial irradiation were used to eradicate disease, but complete remissions were obtained in only 3 patients, and survival of over 1 year in only 2. The remainder died with disease present, less than 5 months from diagnosis.**

SINCE the recognition of the distinctive clinicopathological syndrome of African Burkitt's lymphoma (Burkitt, 1958; O'Connor, 1961) sporadic cases have been reported from most countries, and a series of over 100 children and adolescents has been collected in the United States (Levine *et al.*, 1975).

It is known, however, that the clinical features of the African disease depend on the age at presentation (Burkitt, 1970) and the incidence of the tumour within different tribes (Burkitt and Wright, 1966).

A retrospective study of 9 British children (mean age 7·3 years) showed many similarities in tumour distribution to Africans (Wright, 1966) but more recent reports of American cases have shown marked differences in the frequency of jaw tumours, intra-abdominal and central nervous system (CNS) involvement (Levine *et al.*, 1975) a poorer response to cytotoxic therapy (Ziegler, 1972; Arseneau *et al.*, 1975), and an inability to demonstrate EBV consistently within the tumours (Andersson *et al.*, 1976). There is a much higher frequency of bone marrow infiltration in non-African patients (Levine *et al.*, 1975) and presentation as acute non-myelogenous leukaemia has been reported in 6 cases (mean age 11·7 years) from France (Flandrin *et al.*, 1975).

Because of these marked age-related and geographical differences in the presentation and behaviour of Burkitt's lymphoma, we think it worthwhile to describe the findings of what we believe to be the first reported series of British adults (15 years and over) with Burkitt's lymphoma, with special reference to the clinical features and response to combination cytotoxic therapy, cranial irradiation and maintenance therapy similar to that used for common ALL.

### PATIENTS AND METHODS

The patients, 6 male and 2 female, mean age 24·5 years, were admitted during 1973–74, and comprise less than 5% of cases referred for management of haematopoietic or lymphoreticular malignancies during that period. No patient had received specific therapy prior to admission. One patient (Case 5) had been to a tropical area, visiting central S. America 30 years previously. There were no other relevant points in the social and family histories, and no other serious illnesses. Biopsy material was available in 5 cases, and suitable for electron microscopy (EM) in 3. Touch preparations of tumour from 2 cases, malignant pleural and ascitic fluid from 1, and bone marrow from all cases were stained with May–Grünwald–Giemsa (MGG), periodic acid–Schiff, Sudan black, oil red O, pyronin, and stains for acid phosphatase and α-naphthyl esterase by standard techniques. Tumour cells were examined by EM after fixation in glutaraldehyde and post-fixation in osmium tetroxide and uranyl acetate. Cerebrospinal fluid (CSF) was examined after centrifugation in a cytocentrifuge by MGG and cytochemical stains.

The diagnosis of Burkitt's lymphoma was established in each case, using the morphological, ultrastructural and cytochemical criteria defined by the W.H.O. (Berard *et al.*, 1969). Routine investigations on all patients included a full blood count, liver function tests and estimation of blood urea, electrolytes, uric acid, immunoglobulins and antibodies to EBV. Radiography of the chest and other organs was carried out where clinically indicated, to assess tumour distribution.

### CLINICAL FEATURES

The disease was characterized by an acute onset, with a history of usually less than 8 weeks general ill-health, some weight loss and occasional night sweats. Pain in the jaw occurred in 4 cases and paraesthesiae of the lips and chin in 4. Only 2 patients had sought a dental opinion. Abdominal pain or a change in bowel habit occurred in 5 cases, exploratory laparotomy being performed in 2 for an acute abdomen. Two patients complained of bruising (Cases 5, 8), one of a swelling on the chest wall (Case 4) and one of parotid enlargement (Case 7).

The distribution of tumour at presentation was similar in all patients. Intra-abdominal involvement (7 cases) included infiltration of the terminal ileum, mesenteric and retroperitoneal lymph nodes, liver and kidneys. The spleen was only palpable in 1 patient and there were moderately enlarged peripheral lymph nodes in 3. Cranial nerve (c.n.) lesions were demonstrated in 4 patients, involving c.n.V alone in 3 and c.n. III, V, VI and VII in 1. Small jaw tumours were palpable in 2 cases and demonstrated radiologically in 1 other. Small pleural effusions were noted in 4 cases. Bone marrow infiltration by tumour cells was found in 6 cases—less than 10% of the nucleated cell count in 2 cases, but greater than 70% in the remainder, who also exhibited a leukaemic blood picture (Table I). Moderate anaemia was present in 6 cases and thrombocytopenia (platelets $< 50 \times 10^9$/l) in 3. All patients showed abnormalities of liver function tests, particularly, raised levels of serum aspartate

TABLE I.—*Clinical Features at Presentation of Burkitt's Lymphoma (Disease present +; absent 0)*

| | Case | | | | | | | |
|---|---|---|---|---|---|---|---|---|
| | 1 | 2 | 3 | 4 | 5 | 6 | 7 | 8 |
| Age, Sex | 20 M | 16 M | 36 M | 28 M | 43 M | 21 M | 17 F | 15 F |
| Jaw | + | 0 | + | + | 0 | 0 | 0 | 0 |
| Intra-abdominal | + | + | 0 | + | + | + | + | + |
| CNS | + | 0 | + | + | 0 | + | 0 | 0 |
| Bone marrow | + | 0 | + | 0 | + | + | + | + |
| Circulating tumour cells, $10^9$/l | nil | nil | nil | nil | 0·77 | 57·8 | 0·11 | 1·5 |
| Alk phos., iu/l | 36 | 640 | 53 | 1400 | 112 | 150 | 58 | 105 |
| SGOT, iu/l | 177 | 202 | 38 | 141 | 44 | 110 | 24 | 56 |
| HBD, iu/l | 3000 | 650 | not done | 4000 | 1780 | 3000 | not done | 114 |
| Uric acid, mM | 0·77 | 0·5 | 0·43 | 0·47 | 0·77 | 3·78 | 0·47 | 0·37 |
| EBV titres, 1: | 32 | 128 | 64 | 32 | 16 | 16 | 512 | 128 |

transaminase (SGOT), alkaline phosphatase (Alk. phos.) and hydroxybutyric acid (HBD). Uric dehydrogenase was increased in 7 patients and associated in Case 6 with urate nephropathy and acute renal failure, requiring peritoneal dialysis. There were no significant changes in Ig levels, and all patients had low titres of IgG antibodies to EBV. Relevant haematological and biochemical data are summarized in Table I.

TABLE II.—*Organ Involvement during the Course of the Disease, and Survival*

| Case | 1 | 2 | 3 | 4 | 5 | 6 | 7 | 8 |
|---|---|---|---|---|---|---|---|---|
| Jaw | + | 0 | + | + | 0 | 0 | 0 | 0 |
| Intra-abdominal | + | + | 0 | + | + | + | + | + |
| CNS | + | + | + | + | + | + | + | + |
| Bone marrow | + | + | + | 0 | + | + | + | + |
| Survival (months) | 2 | 5 | 1 | 3 | 5 | 0·5 | >36 | 14 |

TREATMENT AND CLINICAL COURSE

All patients received allopurinol for at least 24 h before commencing combination cytotoxic therapy, which for 7 patients consisted of courses of adriamycin, vincristine, prednisolone and L-asparaginase (Case 6 at reduced dosage), Case 2 receiving courses of cyclophosphamide (CTX), vincristine and prednisolone.

There were 2 early deaths due to septicaemia and thrombocytopenia, but 3 partial and 3 apparently complete remissions were obtained. Lumbar puncture on these 6 cases immediately before changing induction or starting maintenance therapy of CTX, methotrexate (MTX) and 6-mercaptopurine (6-MP), revealed tumour cells in the CSF of 5. Therapeutic CNS treatment in these 5 cases, and prophylaxis in 1, consisted of irradiation to the whole cranium and intrathecal (i.t.) injections of MTX followed by i.t. cytosine arabinoside if infiltration persisted or recurred. This regime caused a rapid but only transient clearing of cells from the CSF in all but Cases 7 and 8. The typical clinical course was resistance to further chemotherapy, with regrowth and spread of tumour, return of malignant cells to the CSF and death 5 months or less from diagnosis. The distribution of tumour and length of survival are shown in Table II. Of the 2 " long-term " survivors, 1 had a bone marrow relapse after 8 months, successfully treated with the same induction therapy, but died in a second relapse at 14 months, and 1 patient remains alive at 36 months, but with tumour cells in the CSF.

DISCUSSION

The pathological diagnosis of Burkitt's lymphoma is still based on a combination of morphological, ultrastructural and cytochemical features, for although both African and non-endemic tumours have been shown to be proliferations of B lymphocytes (Fialkow *et al.*, 1973; Mann *et al.*, 1976) this feature does not clearly differentiate the tumour from many other lymphoid neoplasias. The particular value of Romanowsky-stained tissue imprints in establishing an accurate diagnosis has been stressed previously (Wright, 1967; Levine *et al.*, 1975) and confirmed in this study.

The association of EBV and holoendemic malaria with the African tumours is well known, but the role of EBV in non-endemic cases is less well defined (Andersson *et al.*, 1976). Serum IgG antibodies to EBV were present in all our patients, but material suitable for demonstration of viral genomes within the cells was unfortunately not available. Tumour distribution is similar to that reported in Africans from areas of low tumour incidence (Burkitt and Wright, 1966) and in American cases (Levine *et al.*, 1975), intra-abdominal disease being more prominent than jaw tumours. Seven patients (88%) had bone marrow infiltrated by malignant cells at some stage, compared to 16% of African cases (Bluming, Ziegler and Carbone, 1972) and 31% of American (Banks *et al.*, 1975). Four patients (50%) presented as an acute leukaemia, with clinical features attributable more to a primary haematopoietic disorder, than to terminal dissemination of a lymphoid tumour. This manifestation of Burkitt's lymphoma is rare (Bluming *et al.*, 1972; Flandrin *et al.*, 1975). Presentation with intra-abdominal, CNS, and bone marrow disease, with increased levels of serum enzymes, are features associated in American cases with a median survival of less than 2 months (Arseneau *et al.*, 1975), but 44% of similarly staged African cases survive for 18 months or longer when treated with CTX alone (Ziegler, 1972). Our experience with combination chemotherapy (7 cases receiving adriamycin, vincristine, prenisolone and L-asparaginase), cranial irradiation and i.t. MTX and Ara-C, is similar to results in American cases, in that although 3 apparently complete remissions were achieved 6 cases relapsed, with return or spread of tumour resistant to further chemotherapy, and subsequently died 5 months or less from initial diagnosis. Two patients in remission (both female) received maintenance therapy of CTX, MTX and 6-MP. One relapsed after 8 months, was successfully treated with the same combination chemotherapy, but relapsed again and died 14 months from diagnosis. The second patient remains alive at 36 months, but with CSF involvement. Others have also shown that females survive longer than males (Levine *et al.*, 1975).

It is hoped that a greater awareness of the occurrence of Burkitt's lymphoma in British adults, especially the combination of intra-abdominal disease with c.n. lesions, will lead to further insights into the pathogenesis of the disorder and better therapeutic measures.

We wish to thank Dr M. E. J. Beard of St Bartholomew's Hospital for his advice on the haematology of these patients, Professor D. H. Wright (Southampton University) for reviewing Case 7, Joan N. B. Edwards of the Virus Reference Laboratory, Colindale for performing EBV antibody tests, and Mrs L. Brown for performing the cytochemical tests. The patients were admitted under the care of the late Professor G. Hamilton Fairley.

### REFERENCES

ANDERSSON, M., KLEIN, G., ZIEGLER, J. L. & HENLE, W. (1976) Association of Epstein–Barr Viral Genomes with American Burkitt Lymphoma. *Nature, Lond.*, **260**, 357.

ARSENEAU, J. C., CANELLOS, G. P., BANKS, P. M., BERARD, C. W., GRALNICK, H. R. & DE VITA, V. T. (1975) American Burkitt's Lymphoma: A Clinicopathologic Study of 30 Cases. I. Clinical Factors Relating to Prolonged Survival. *Am. J. Med.*, **58**, 314.

BANKS, P. M., ARSENEAU, J. C., GRALNICK, H. R., CANELLOS, G. P., DE VITA, V. T. & BERARD, C. W. (1975) American Burkitt's Lymphoma: A Clinicopathologic Study of 30 Cases. II. Pathologic Correlations. *Am. J. Med.*, **58**, 322.

BERARD, C. W., O'CONNOR, G. T., THOMAS, L. B. & TORLONI, H. (1969) Histopathological Definition of Burkitt's Tumour. *W.H.O. Bull.*, **40**, 601.

BLUMING, A. Z., ZIEGLER, J. L. & CARBONE, P. P. (1972) Bone Marrow Involvement in Burkitt's Lymphoma: Results of a prospective study. *Br. J. Haematol.*, **22**, 369.

BURKITT, D. P. (1958) A Sarcoma Involving the Jaws in African Children. *Br. J. Surg.*, **46**, 218.

BURKITT, D. P. (1970) In *Burkitt's Lymphoma.* Eds D. P. Burkitt and D. H. Wright. Edinburgh and London: Livingstone.

BURKITT, D. P. & WRIGHT, D. H. (1966) Geographical and Tribal Distribution of the African Lymphoma in Uganda. *Br. med. J.*, i, 569.

FIALKOW, P. J., KLEIN, E., KLEIN, G., CLIFFORD, P. & SINGH, S. (1973) Immunoglobulin and Glucose-6-Phosphate Dehydrogenase as Markers of Cellular Origin in Burkitt Lymphoma. *J. exp. Med.*, **138**, 89.

FLANDRIN, G., BROUET, J. C., DANIEL, M. T. & PREUD'HOMME, J. L. (1975) Acute Leukemia with Burkitt's Tumor Cells: A Study of Six Cases with Special Reference to Lymphocyte Surface Markers. *Blood*, **45**, 183.

LEVINE, P. H., CHO, B. R., CONNELLY, R. R., BERARD, C. W., O'CONNOR, G. T., DORFMAN, R. F., EASTON, J. M. & DE VITA, T. V. (1975) The American Burkitt Lymphoma Registry: A Progress Report. *Ann. int. Med.*, **83**, 31.

MANN, R. B., JAFFE, E. S., BRAYLAN, R. C., NANBA, K., FRANK, M. M., ZIEGLER, J. L. & BERARD, C. W. (1976) Non-endemic Burkitt's Lymphoma. A B-cell Tumor Related to Germinal Centres. *N. Engl. J. Med.*, **295**, 685.

O'CONNOR, G. T. (1961) Malignant Lymphoma in African Children. II. A Pathological Entity. *Cancer, N.Y.*, **14**, 270.

WRIGHT, D. H. (1966) Burkitt's Tumour in England: A Comparison with Childhood Lymphosarcoma. *Int. J. Cancer*, **1**, 503.

WRIGHT, D. H. (1967) Burkitt's Tumor and Childhood Lymphosarcoma. *Clin. Pediatrics*, **6**, 116.

ZIEGLER, J. L. (1972) Chemotherapy of Burkitt's Lymphoma. *Cancer, N.Y.*, **30**, 1534.